# The Efficacy and Safety of Oral Spironolactone in the Treatment of Female Pattern Hair Loss: A Systematic Review and Meta-Analysis

**DOI:** 10.7759/cureus.43559

**Published:** 2023-08-16

**Authors:** Majed Aleissa

**Affiliations:** 1 Department of Dermatology, King Abdulaziz Medical City Riyadh, Riyadh, SAU

**Keywords:** safety, adverse effects, efficacy, spironolactone, androgenetic alopecia

## Abstract

Oral spironolactone has been proposed as a potential treatment for hair loss due to its antiandrogenic properties. However, the efficacy and safety of spironolactone for treating hair loss are not well-established.

The objective of this study was to conduct a systematic review of the current literature on the use of oral spironolactone in female pattern hair loss.

We conducted a systematic review and meta-analysis of randomized controlled trials and observational studies that assessed the efficacy and safety of oral spironolactone for treating hair loss. We searched for eligible papers in PubMed, Web of Science (ISI), Embase, and Scopus. All analyses were done using R software version 4.2.3 (R Foundation for Statistical Computing, Vienna, Austria).

The overall rate of improved hair loss was 56.60%, with a higher rate of improvement (65.80%) observed in the combined therapy group compared to the monotherapy group (43.21%). However, there was significant heterogeneity in the efficacy outcomes, and hair loss did not improve or showed a modest improvement in 37.80% of all patients. The rates of adverse events reported in at least two studies were scalp pruritus or increased scurf (18.92%), menstrual disorders (11.85%), facial hypertrichosis (6.93%), and drug discontinuation (2.79%). The overall adverse events rate was 3.69%, but there was significant heterogeneity in the rates of different adverse events.

In conclusion, the present study suggests that spironolactone is an effective and safe treatment option for hair loss. However, further research is needed to fully understand the heterogeneity of treatment response and adverse events and identify factors that may predict treatment response.

## Introduction and background

Female pattern hair loss, also known as female androgenetic alopecia, is a common form of hair loss in women that affects up to 50% of women over the age of 50 [1]. The condition is characterized by a progressive thinning of hair on the scalp, typically in a diffuse pattern, and can result in significant psychological distress and reduced quality of life for affected individuals [2, 3].

Currently, there are limited effective treatment options for female pattern hair loss, and many of the available treatments, such as topical minoxidil and oral finasteride, have limitations in terms of efficacy, tolerability, and safety [1]. This has led to increasing interest in the potential role of spironolactone, an aldosterone antagonist with antiandrogenic effects, in the management of female pattern hair loss.

Spironolactone is a medication commonly used in the treatment of hypertension and heart failure, but it has also been shown to have antiandrogenic effects, which may be beneficial in the treatment of female pattern hair loss [4]. The antiandrogenic effects of spironolactone are believed to be mediated through its ability to inhibit the binding of dihydrotestosterone (DHT) to androgen receptors, thus reducing the production of sebum and the miniaturization of hair follicles [4]. Several studies have investigated the use of spironolactone in the treatment of female pattern hair loss, with promising results. Spironolactone was effective in improving hair density and reducing hair loss in women with female pattern hair loss, with no significant adverse effects reported [5, 6].

In this paper, we will conduct a systematic review of the current literature on the use of oral spironolactone in female pattern hair loss, including its efficacy and potential side effects. We hope that this review will provide a comprehensive analysis of the existing evidence on the use of spironolactone in female pattern hair loss and help guide future studies in this area.

## Review

Methods

Search Strategy and Study Selection

We followed the recommendations of the Preferred Reporting Items for Systematic Reviews and Meta-Analyses (PRISMA) and MOOSE (Meta-analyses Of Observational Studies in Epidemiology) checklists for conducting systematic reviews and meta-analyses. On March 26, 2023, we searched for eligible papers in PubMed, Web of Science, Embase, and Scopus. We tailored the search strategy based on each database; the detailed strategy is provided in Appendix 1.

Study selection was made using the PICO (population, intervention, control, and outcomes) framework: participants were patients with established androgenic alopecia diagnosis in females, and the intervention was oral spironolactone. The primary efficacy outcome was the rate of improved hair loss, while the secondary outcomes were the rates of no improvement or modest improvement and hair loss worsening. The primary safety outcome was the rate of adverse events associated with spironolactone treatment. We included all original studies satisfying the pre-defined criteria. We excluded non-English papers, studies using topical spironolactone, animal studies, non-original studies, case reports, and case series with less than ten patients. The screening was done in two stages - title and abstract screening, followed by a full-text one. Both stages were done by two reviewers, with the third one resolving any conflicts.

Two authors used the pre-designed Excel sheet (Microsoft Corporation, Redmond, USA) to extract all relevant data points, with the senior author doing quality control or resolving any disputes. The combined therapy was defined as the use of oral spironolactone and minoxidil, while the monotherapy was defined as using oral spironolactone alone, regardless of the dose used. The risk of bias was assessed by two reviewers using the Newcastle-Ottawa scale for the assessment of the quality of nonrandomized studies [7] and used the revised tool for assessing the risk of bias in randomized trials for randomized controlled trials (RCTs) [8]. In case of any disagreements, a third senior author would resolve them.

Statistical Analysis

All outcomes reported in at least two studies were included in the meta-analysis. We calculated prevalence rates and their corresponding 95% confidence intervals (CI) using the generalized linear mixed models with the logit link. The random-effects model was employed to pool the data due to methodological differences violating the common-effects assumption. Heterogeneity was assessed using the Q statistic and I2 test, where I2>50% or P-value <0.05 was considered significant. For the worsening of hair loss outcome, we used double arcsine transformation due to the presence of two zero events [9]. Publication bias using funnel plots was not possible due to the small number of included studies (<10) [10,11]. All analyses were done using R software version 4.2.3 (R Foundation for Statistical Computing, Vienna, Austra).

Search results

**Figure 1:**
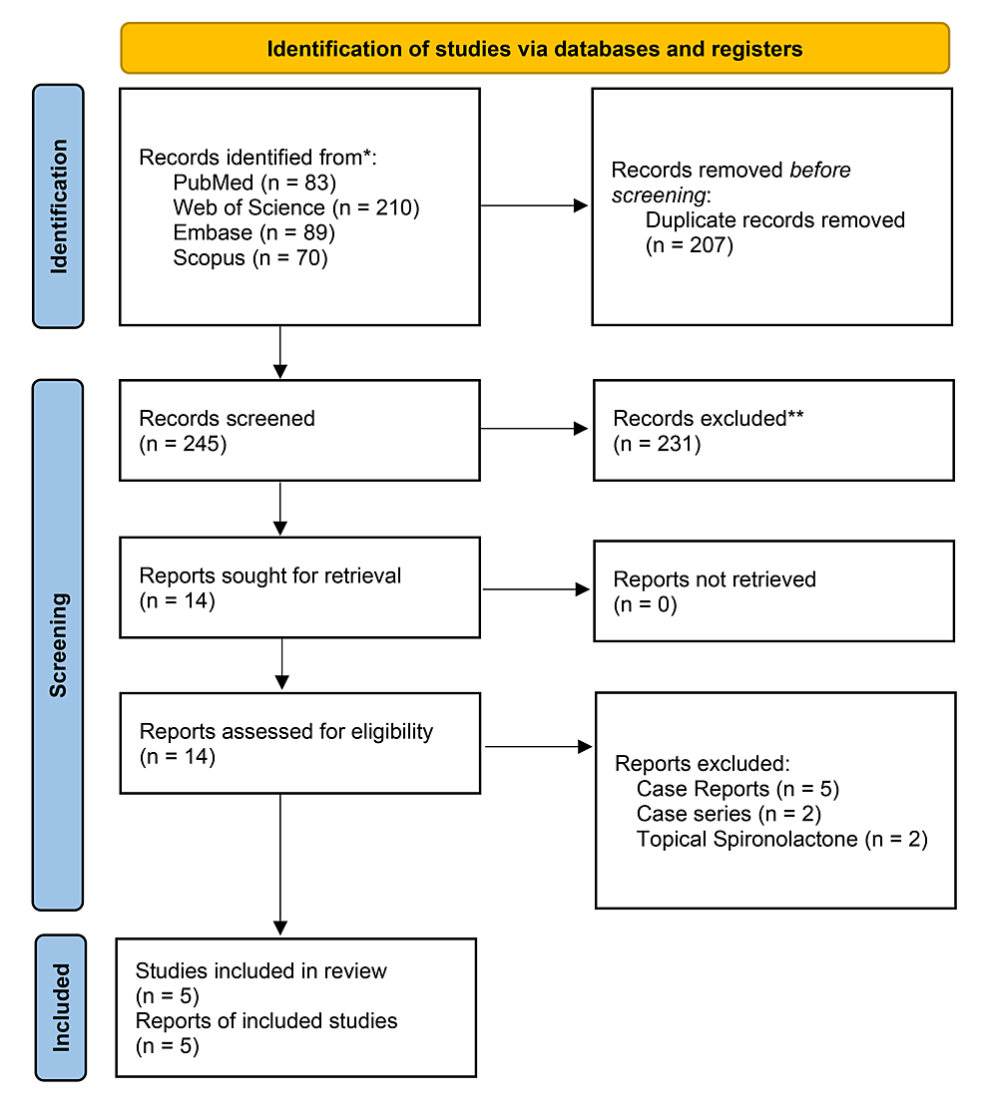
PRISMA flow diagram of the study process PRISMA: Preferred Reporting Items for Systematic Reviews and Meta-Analyses

We initially retrieved 452 records, of which 207 were duplicates, to end up with 245 studies for the title and abstract screening. After excluding 231 studies in the first screening stage, 14 papers passed to full-text screening, to end up finally with five studies [5,12-15]. The search and screening process is summarized in Figure 1.

Study characteristics and risk of bias

The included studies have variable study designs such as prospective, retrospective, survey study, RCT, and a large case series. The number of patients ranged from 19 to 115, and the age ranged from 12 to 88 years in the included studies. The shortest treatment duration was six months, and the longest was over a year. The dosing regimen and treatments used were also variable across studies (Table 1). The risk of bias assessment results are summarized in the supplementary tables given in Appendix 2.

**Table 1:** Summary of the included studies SPT: spironolactone; MX: topical minoxidil, FPHL: female pattern hair loss

Author, Year	Study design	Total Patients	Age range (Years)	Duration of treatment	Treatment regimen	Drug Comparison	Adverse events %	Outcome assessments	Conclusion
Sinclair, 2005 [12]	Prospective study	80	12–79	Mean 16 months	Oral SPT 200 mg day	Cyproterone acetate; 50 mg daily continuously Post menopause and 100 mg daily for 10 days each month together with a combination oral contraceptive pill Perimenopause	NA	Photography	80% of women receiving oral antiandrogens showed no progression or improvement of their FPHL. There was no predictor of response except High midscalp clinical grade.
Sinclair, 2018 [13]	Case series	100	18-80	12 months	Oral MX 0.25 mg and SPT 25 mg	NA	urticaria (2), Postural hypotension (2), and hypertrichosis (4)	Hair shedding score and Hair loss severity score	daily capsules that contain SPT 25 mg and MX 0.25 mg seems to be safe and effective as well in FPHL treatment.
Famenini, 2015 [15]	Retrospective study	19	NA	NA	SPT (mean dose= 110mg)	NA	Side effects were consistent with product label.	Patient and physician assessments and medical history review	74.3% of females using SPT reported improvement or stabilization of their FPHL.
	Survey study	20	20–88					Self-reported	
Burns, 2020 [14]	Retrospective study	79	21–79	ranged: 6 months in 31 (39%); 1 year in 23 (29%); and >1 year: 22 (28%)	Oral SPT with mean dose of 100mg (range 25–200mg) daily. Some patient used SPT monotherapy and others used Concomitant therapies started with SPT, including topical MX, low-level laser light device, and iron supplementation.	NA	Breast tenderness (1.3), Dizziness/Light headedness (16.5), Self-resolving hyperkalemia (1.3), Menstrual spotting (2.5), Nausea (2.5), Increased urination (2.5), Rash (2.5), and others (8.9)	Sinclair Score	Current treatments available for FPHL right now are limited, especially oral medications that promote increased compliance and convenience of administration. This study offers more proof that SPT, whether used as monotherapy or adjunct therapy, is a successful and well-tolerated treatment choice for FPHL.
Liang, 2022 [5]	RCT	115	18–45	24 weeks	Oral SPT of 80–100 mg/day and 1 ml of topical MX 5% once daily.	Group 1:1 mL of topical MX tincture 5% once daily; Group 2: micro-needling treatments with the delivery of 5% MX every 2 weeks and 1 mL of topical 5% MX once daily.	Menstrual disorder (40.5), Scalp pruritus (21.6), Increased scurf (16.2), Facial hypertrichosis (13.5), Trichomadesis aggravating (10.8), Palpitation (8.1), Edema of the limbs (2.7), Urticaria (2.7), and Hyperkalemia (2.7)	Ultrasound bio-microscopy, Photography, and Dermoscopy	Topical MX combined with micro-needling is a better than either MX plus oral SPT or MX alone for the treatment of mild-to-moderate FPHL.

Efficacy and safety

**Figure 2:**
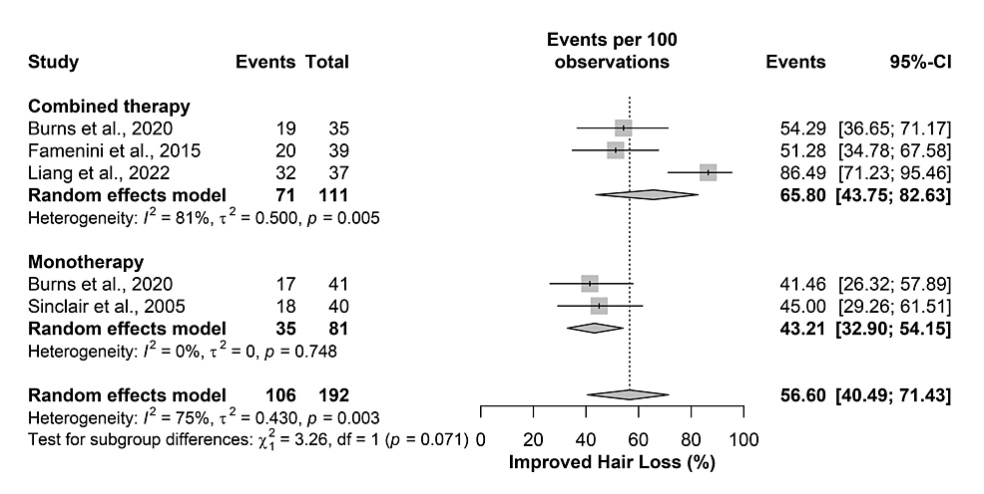
The overall rate of improved hair loss [12-15]

Four studies of 192 patients assessed changes in hair loss following oral spironolactone treatment. The overall rate of improved hair loss was 56.60% (95% CI=40.49-71.43), 65.80% (95% CI=43.75-82.63) in the combined therapy, and 43.21% (95%CI=32.90-54.15) in the monotherapy. One study used the Women’s Alopecia Severity index [15], the Sinclair grading score in three [5, 13, 14], and the Ludwig scale and the mid-scalp clinical grading scale in one [12]. However, there was a significant overall heterogeneity (I2=75%; P-value=0.003), and monotherapy and the combined therapy subgroup (I2=81%; P-value=0.005) (Figure 2).

**Figure 3:**
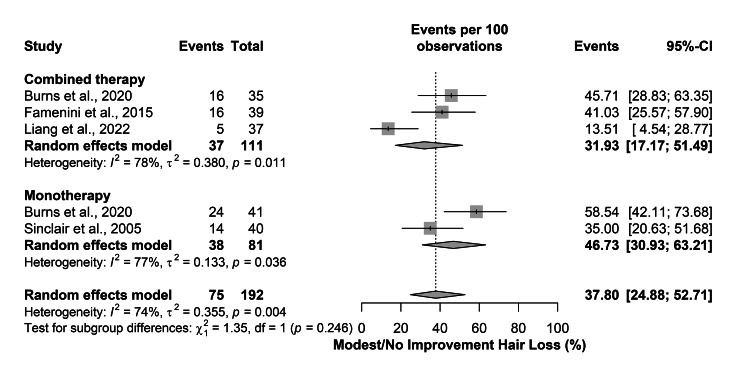
Subgroup analysis of the overall rate of improved hair loss [12-15]

The hair loss did not improve or showed a modest improvement in 37.80% (95% CI=24.88-52.71) of all patients. Further subgroup analysis showed that the rate was 31.93% (95% CI=17.17-51.49) in the combined therapy and 46.73% (95% CI=30.93-63.21) in the monotherapy. Nevertheless, heterogeneity was present in both overall (I2=74%; P-value=0.004) and subgroup estimates (Figure 3).

Hair loss worsened in 3.64% (95% CI=0.16-9.90) of all treated patients, in 3.45% (95% CI=0.01-10.37) of those on combined therapy, and in 3.97% (95% CI=0.00-23.81) of those on monotherapy. Heterogeneity was significant in the overall estimate (I2=64%; P-value=0.025) and within the monotherapy subgroup (I2=86%; P-value=0.007). In all efficacy outcomes, the rates were comparable between combined therapy and monotherapy groups. 

**Figure 4:**
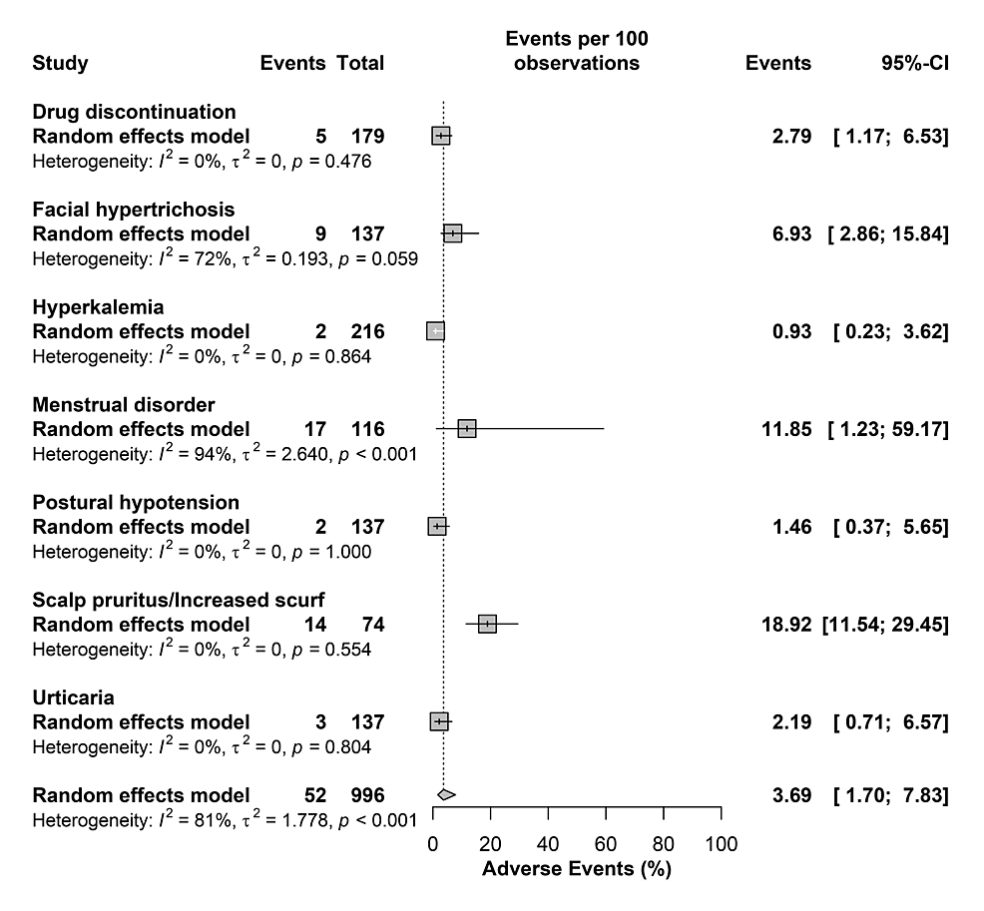
Prevalence of adverse events [12-15]

For adverse events reported in at least two studies, scalp pruritis or increased scurf were the most frequently encountered at 18.92% (95% CI=11.54-29.45), followed by menstrual disorders, facial hypertrichosis, and drug discontinuation with 11.85% (95% CI=1.23-59.17), 6.93% (95% CI=2.86-15.84), and 2.79% (95% CI=1.17-6.53), respectively. The overall adverse events rate was 3.69% (95% CI=1.70-7.83); however, there was significant heterogeneity in the rates of different adverse events (I2=81%; P-value<0.001) (Figure 4).

Discussion

Our systematic review and meta-analysis of available evidence aimed to evaluate the efficacy and safety of oral spironolactone for treating hair loss. The results of this study suggest that oral spironolactone may be an effective treatment for hair loss, especially when combined with other therapies. However, the significant heterogeneity observed in the efficacy outcomes indicates that the treatment response may vary across individuals. The rates of adverse events reported in this study are consistent with previous reports, and the overall adverse events rate is low. The most frequently encountered adverse event was scalp pruritus or increased scurf, which is a minor and manageable side effect. Menstrual disorders, facial hypertrichosis, and drug discontinuation were also reported in some studies. The overall adverse events rate was 3.69%, but there was significant heterogeneity in the rates of different adverse events. These findings suggest that spironolactone treatment may carry a risk of adverse events and should be used with caution, especially in patients with a history of menstrual disorders or other hormonal imbalances.

The results of this study are consistent with previous literature. A previous systematic review reported that spironolactone was effective in reducing hair loss in women with androgenetic alopecia [16]. In previous research conducted by Burns [14] and Sinclair [13], the efficacy of spironolactone in treating androgenetic alopecia was found to be significantly better with a 12-month treatment compared to a 6-month treatment.

Many other studies and case reports have also reported better efficacy of this combined therapy for androgenetic alopecia. For example, a 53-year-old woman with diagnosed androgenetic alopecia was successfully treated with a combination of daily spironolactone and topical minoxidil, which had an additive effect on hair regrowth [17]. Furthermore, in a similar case series of six adolescents found an evident improvement in five of them using the combination of oral minoxidil and spironolactone [18].

Limitations of this meta-analysis include the small sample size (413 patients) and the significant heterogeneity observed in the efficacy outcomes and adverse event rates. In addition, the heterogeneity in study designs, dosing regimen, duration, and how other agents are combined would limit the generalizability of the findings. Further studies with larger sample sizes and more rigorous study designs are needed to confirm these findings and determine the optimal dosing and duration of oral spironolactone treatment for hair loss.

## Conclusions

Oral spironolactone may be an effective and safe treatment for hair loss, especially when combined with other therapies. Clinicians should consider the potential benefits and risks of this treatment option when managing patients with hair loss. Despite the promising results, there is still a need for further research to establish the optimal dosing regimens and treatment duration for spironolactone in female pattern hair loss with determining the safety and tolerability of spironolactone in this population.

## Appendices

Appendix 1: search strategy

PubMed

('spironolactone' [MeSH] OR 'spironolactone' OR 'spironolactones') AND ("androgenic alopecia"[MeSH Terms] OR 'androgenic alopecia'[tw] OR 'female pattern hair loss'[tw] OR 'hormonal hair loss'[tw] OR 'androgenetic alopecia'[tw] OR 'androgenic hair loss'[tw] OR 'androgenetic hair loss'[tw] OR 'hormonal alopecia'[tw])

Results: 83

Web of Science

 (TS= (“androgenic alopecia”) OR TS=(“alopecia”) OR TS=("female pattern hair loss") OR TS=

("androgenetic alopecia") OR TS= (“hormonal hair loss") OR TS=("androgenic hair loss") OR

TS=(“hormonal alopecia"))

 (TS=(spironolactone OR spironolactones))

 #1 AND #2

Results: 210

Embase

 ('spironolactone' or 'spironolactone' or 'spironolactones').ab,ti.

 ("androgenic alopecia" or 'androgenic alopecia' or 'female pattern hair loss' or 'hormonal hair loss' or 'androgenetic alopecia' or 'androgenic hair loss' or 'androgenetic hair loss' or 'hormonal alopecia').ab,ti.

 #1 AND #2

Results: 89

Scopus

 ( TITLE-ABS ( 'spironolactone'  OR  'spironolactone'  OR  'spironolactones' ) ) 

 ( TITLE-ABS ( "androgenic alopecia"  OR  "androgenic alopecia"  OR  "female pattern hair loss"  OR  "hormonal hair loss"  OR  "androgenetic alopecia"  OR  "androgenic hair loss"  OR  "androgenetic hair loss"  OR  "hormonal alopecia" ) ) 

 #1 AND #2

Results: 70

Appendix 2: supplementary tables

**Table 2:** Risk of bias of non-RCTs assessed by the Newcastle-Ottawa Scale RCT: randomized controlled trial; NOS: Newcastle-Ottawa Scale

	Selection	Comparability	Exposure/Outcome	NOS Score
Sinclair, 2005	★★★	★★	★★★	8
Sinclair, 2018	★★	★	★★★	6
Famenini, 2015	★	★★	★★★	6
Burns, 2020	★★	★★	★★★	7

**Table 3:** Risk of bias of RCTs assessed by Cochrane Collaboration’s risk of bias assessment tool RCT: randomized controlled trial

	Random Sequence Generation	Allocation Concealment	Blinding Of Participants And Personnel	Blinding Of Outcome Assessment	Incomplete Outcome or Data	Selective Reporting	Other Bias
Liang, 2022	Low	Unclear	High	Low	Low	Low	Low
